# A Mouse Model of Acrodermatitis Enteropathica: Loss of Intestine Zinc Transporter ZIP4 (Slc39a4) Disrupts the Stem Cell Niche and Intestine Integrity

**DOI:** 10.1371/journal.pgen.1002766

**Published:** 2012-06-21

**Authors:** Jim Geiser, Koen J. T. Venken, Robert C. De Lisle, Glen K. Andrews

**Affiliations:** 1 Department of Biochemistry and Molecular Biology, University of Kansas Medical Center, Kansas City, Kansas, United States of America; 2 Department of Molecular and Human Genetics, Baylor College of Medicine, Houston, Texas, United States of America; 3 Department of Anatomy and Cell Biology, University of Kansas Medical Center, Kansas City, Kansas, United States of America; CHU de Nantes Hôtel-Dieu, France

## Abstract

Mutations in the human *Zip4* gene cause acrodermatitis enteropathica, a rare, pseudo-dominant, lethal genetic disorder. We created a tamoxifen-inducible, enterocyte-specific knockout of this gene in mice which mimics this human disorder. We found that the enterocyte *Zip4* gene in mice is essential throughout life, and loss-of-function of this gene rapidly leads to wasting and death unless mice are nursed or provided excess dietary zinc. An initial effect of the knockout was the reprogramming of Paneth cells, which contribute to the intestinal stem cell niche in the crypts. Labile zinc in Paneth cells was lost, followed by diminished Sox9 (sex determining region Y-box 9) and lysozyme expression, and accumulation of mucin, which is normally found in goblet cells. This was accompanied by dysplasia of the intestinal crypts and significantly diminished small intestine cell division, and attenuated mTOR1 activity in villus enterocytes, indicative of increased catabolic metabolism, and diminished protein synthesis. This was followed by disorganization of the absorptive epithelium. Elemental analyses of small intestine, liver, and pancreas from *Zip4*-intestine knockout mice revealed that total zinc was dramatically and rapidly decreased in these organs whereas iron, manganese, and copper slowly accumulated to high levels in the liver as the disease progressed. These studies strongly suggest that wasting and lethality in acrodermatitis enteropathica patients reflects the loss-of-function of the intestine zinc transporter ZIP4, which leads to abnormal Paneth cell gene expression, disruption of the intestinal stem cell niche, and diminished function of the intestinal mucosa. These changes, in turn, cause a switch from anabolic to catabolic metabolism and altered homeostasis of several essential metals, which, if untreated by excess dietary zinc, leads to dramatic weight loss and death.

## Author Summary

Loss-of-function of the zinc transporter ZIP4 in the mouse intestine mimics the lethal human disease acrodermatitis enteropathica. This is a rare disease in humans that is not well understood. Our studies demonstrate the paramount importance of ZIP4 in the intestine in this disease and reveal that a root cause of lethality is disruption of the intestine stem cell niche and impaired function of the small intestine. This, in turn, leads to dramatic weight loss and death unless treated with exogenous zinc.

## Introduction

The rare pseudo-dominant genetic disease acrodermatitis enteropathica (AE) is thought to be caused by the inefficient absorption of dietary zinc [1]. AE occurs at a frequency 1 in 500,000 and symptoms in humans usually develop soon after birth in bottle fed infants or after weaning in breast fed infants. The triad of alopecia, eczematous dermatitis and diarrhea are classic symptoms of AE in humans and patients experience growth retardation and a myriad of symptoms of severe zinc deficiency which eventually lead to death unless treated by exogenous zinc [2].

In 2002, the AE gene was mapped to human chromosomal region 8q24.3 and the *Zip4* (Slc39a4) locus [3], [4]. Currently, over 32 mutations or variants of ZIP4 have been reported [5]. Missense and nonsense mutations as well as deletions or rearrangements of the gene have all been reported, and hypomorphic as well as complete loss-of-function alleles have been identified.

Recent studies have shed light on the mechanisms of *Zip4* regulation and function [6]. Mouse *Zip4* is most actively expressed in tissues involved in the absorption of dietary or maternal zinc, but also shows high level expression in other cell-types (e.g. pancreatic islet cells, brain capillaries), and low level expression in other tissues (e.g. liver, kidney) and some cultured cells. *Zip4* expression is regulated by cell-specific transcription as well as by multiple posttranscriptional mechanisms in response to zinc availability. ZIP4 protein is at the apical surface of enterocytes and endoderm cells when zinc is deficient, due to increased mRNA and protein stability. During zinc deficiency ZIP4 undergoes processing by removal of the extracellular amino-terminus. In contrast, in the presence of normal levels of zinc *Zip4* mRNA is unstable and the protein is internalized and rapidly degraded.

ZIP4 function is critical during periods of rapid growth when zinc requirements are high but this zinc transporter also has important functions when zinc is replete. *Zip4* is aberrantly expressed in many cancers [7], [8]. Knockdown of ZIP4 can slow cell cycle and cell migration in mouse Hepa cells and ZIP4 functions to reduce apoptosis and enhance cell cycle in hepatomas and to enhance pancreatic tumor growth in nude mice [7], [8]. Many recent studies have demonstrated that zinc can modulate signal transduction cascades [9].

The essential function of ZIP4 in zinc homeostasis is confirmed in *Zip4*-intestine knockout mice. Homozygous *Zip4*-intestine knockout mouse embryos die soon after implantation because this gene is also actively expressed in the visceral endoderm which surrounds the developing mouse embryo and which serves a nutrient uptake function before development of the placenta. The visceral yolk sac is a more vestigial organ in humans and apparently contributes little to providing nutrients for the embryo, explaining why loss of ZIP4 in humans is apparently not embryonic-lethal. Interestingly, heterozygous *Zip4*-intestine knockout mouse embryos are significantly underrepresented in the population at parturition. These heterozygous embryos display an array of developmental defects including exencephalia, anophthalmia, and severe growth retardation. About 22% of the *Zip4*-heterozygous mice that survive to weaning age are severely growth retarded and display anopia, anopthalmia, hydrocephalus and heart defects, among other abnormalities [10]. Mice heterozygous for *Zip4* knockout are hypersensitive to zinc deficiency. Thus, haploinsufficiency of *Zip4* may contribute to growth retardation in humans, an effect that is probably exacerbated by zinc deficiency and/or by modifier genes. Mutations in genes essential for posttranscriptional regulation of ZIP4 may also cause AE although this has not been demonstrated. The finding of *Zip4* haploinsufficiency defines AE as a pseudo-autosomal dominant trait.

Given that a global knockout of *Zip4* is embryonic lethal in mice, unlike it is in humans, we sought to develop a better mouse model of AE. To that end we created mice with floxed *Zip4* genes and bred them with mice that express an ErtCre fusion protein driven by the *villin* promoter [11] specifically in intestine enterocytes. Using this tamoxifen-inducible *Zip4*-enterocyte knockout model we provide evidence that intestine expression of *Zip4* is essential for the growth and viability unless mice are supplied with excess zinc and that an absence of ZIP4 in the intestine appears to closely mimic the AE phenotype in humans. Moreover we provide evidence that an absence of ZIP4 only in the intestine leads to a rapid switch from anabolic to catabolic metabolism in the animal, to tissue-specific dysregulation of other essential metals and alterations in gene expression. These phenotypes appear to reflect compromised Paneth cell functions which lead to disruption of the intestinal stem cell niche ultimately resulting in loss of intestinal integrity and diminished nutrient uptake.

## Results

### Knocking out the *Zip4* gene in the intestinal epithelium

**Figure 1:**
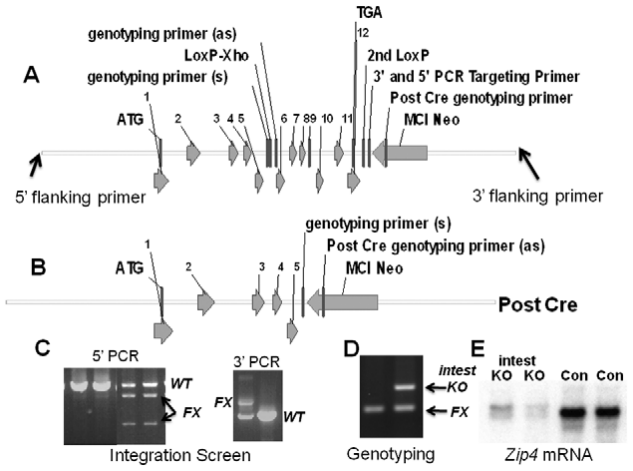
Structures of the pre- and post-Cre floxed mouse *Zip4* gene and integration and genotyping screen designs. (A) The mouse *Zip4* gene was captured using gap-repair and then manipulated using recombineering and the galK selection system. Exons (1–12) are indicated, as are the positions of *LoxP* sites (intron 5 and downstream of exon 12), the *mc1-neomycin* (*MC1-Neo*) cassette and the primers used to screen for integration and genotyping. (B) The structure of the *Zip4* gene after Cre recombination is shown. The floxed *Zip4* gene was targeted into E14 ES cells. (C) Properly targeted ES cells were identified by long range PCR using flanking and internal primers. PCR products from the control (*Con*) and floxed (*FX*) alleles are indicated. *XhoI* cleavage was used to differentiate between the floxed and wild-type alleles in the 5′ PCR screen whereas the 3′ PCR screen yielded the predicted larger product from the floxed allele. Targeted ES cells were used to generate mice homozygous for the floxed *Zip4* allele. (D) Mice were genotyped by PCR amplification of the intron 5 region containing the *LoxP* site. The PCR product from homozygous mice before Cre-induced recombination is shown (left lane). A *vil-CreERT2* transgene was bred into mice homozygous for the floxed *Zip4* gene to allow for tamoxifen induction of Cre activity specifically in the intestinal epithelium. DNA from the intestine of tamoxifen injected mice was amplified by PCR to examine the extent of recombination of the floxed gene (right lane). PCR products from the recombined floxed intestine allele (*intest KO*) and the remaining floxed alleles (*FX*) shown. About 50% of the cells in the intestine are epithelial which is reflected in the extent of recombination shown. (E) Northern blot detection of intestine *Zip4* mRNA in control (Con) and *Zip4*-intestine knockout mice (intest KO) 3 days after initiation of the knockout.

To enable tissue-specific deletion of the mouse *Zip4* gene a targeting construct was created which contained a *LoxP* site flanked by an *XhoI* restriction site in intron 5, and a LoxP site just downstream of the last exon (exon 12), followed by an mc1-Neomycin cassette (Figure 1). This construct was targeted in E14 embryonic stem (ES) cells and cells with proper integration of the floxed *Zip4* gene were then identified by long-range PCR using primers outside of the engineered targeting construct coupled with overlapping internal primers (Figure 1A). The 5′ integration screen (Figure 1C) amplified a 7.35 kb product from the wild-type and the floxed alleles and cleavage of the floxed allele with *XhoI* yielded the predicted 5.2 and 2.1 kb restriction fragments indicative of proper insertion of the floxed allele into one of the endogenous *Zip4* alleles. This was confirmed by the 3′ integration screen which yielded a 3.4 kb PCR product from the floxed allele and a 2.1 kb PCR product from the wild-type allele. Targeted E14 ES cells were used to create chimeric mice by blastocyst injection and agouti offspring from the chimeric mice were genotyped using primers which flank the LoxP-XhoI insertion site in intron 5 (Figure 1A). Genotyping PCR yielded a 227 bp product from the floxed allele, which could be cleaved by *XhoI*, and a 187 bp product from the wild-type allele (Figure 1D).

To enable tissue-specific deletion of the floxed *Zip4* gene specifically in the intestinal epithelium, *Zip4* ^FX/FX^ mice were crossed with mice that express an estrogen receptor-Cre recombinase fusion protein under control of the *villin* promoter [11]. *Vil-Cre-ERT2* is expressed only in the intestinal epithelium and visceral endoderm. The Cre-ERT2 fusion protein resides in the cytoplasm and is driven to the nucleus by tamoxifen which allows for temporal control of the *Zip4* gene deletion [11]. Mice heterozygous for *vil-Cre-ERT2* and homozygous *Zip4* ^FX/FX^ were crossed with *Zip4^FX/FX^* mice to yield 50% offspring with *Zip4^FX/FX^*: *vil-Cre-ERT2* alleles and 50% with *Zip4^FX/FX^* alleles. The latter provided age and genetically matched controls for all of our experiments and are labeled as control (Con) mice in the figures. Tamoxifen injections resulted in recombination of approximately 50% of the floxed *Zip4* genes in the small intestine of recently weaned *Zip4^FX/FX^*: *vil-Cre-ERT2* mice (Figure 1D: intest KO). This is as expected since the epithelial cells represent about half of the cells in the small intestine. The extent of loss of *Zip4* mRNA however provides a suitable assay for the extent of recombination of the floxed *Zip4* gene in the epithelium since the expression of this gene in the intestine is restricted to the epithelium. Three injections of tamoxifen resulted in a dramatic, if not complete loss of intestine *Zip4* mRNA (Figure 1E: intest KO) which suggests that the vast majority, if not all, of the floxed enterocyte *Zip4* genes had undergone recombination. ZIP4 protein in the intestine of mice fed a zinc-adequate diet is present at only low levels but this mRNA and protein are stabilized when zinc is deficient. ZIP4 protein and mRNA are rapidly turned-over when dietary zinc is restored. The loss of *Zip4* mRNA is expected to be followed by the turn-over of ZIP4 protein within a few hours [12].

### The intestine *Zip4* gene controls growth and viability but knockout mice can be rescued by nursing or excess dietary zinc

**Figure 2:**
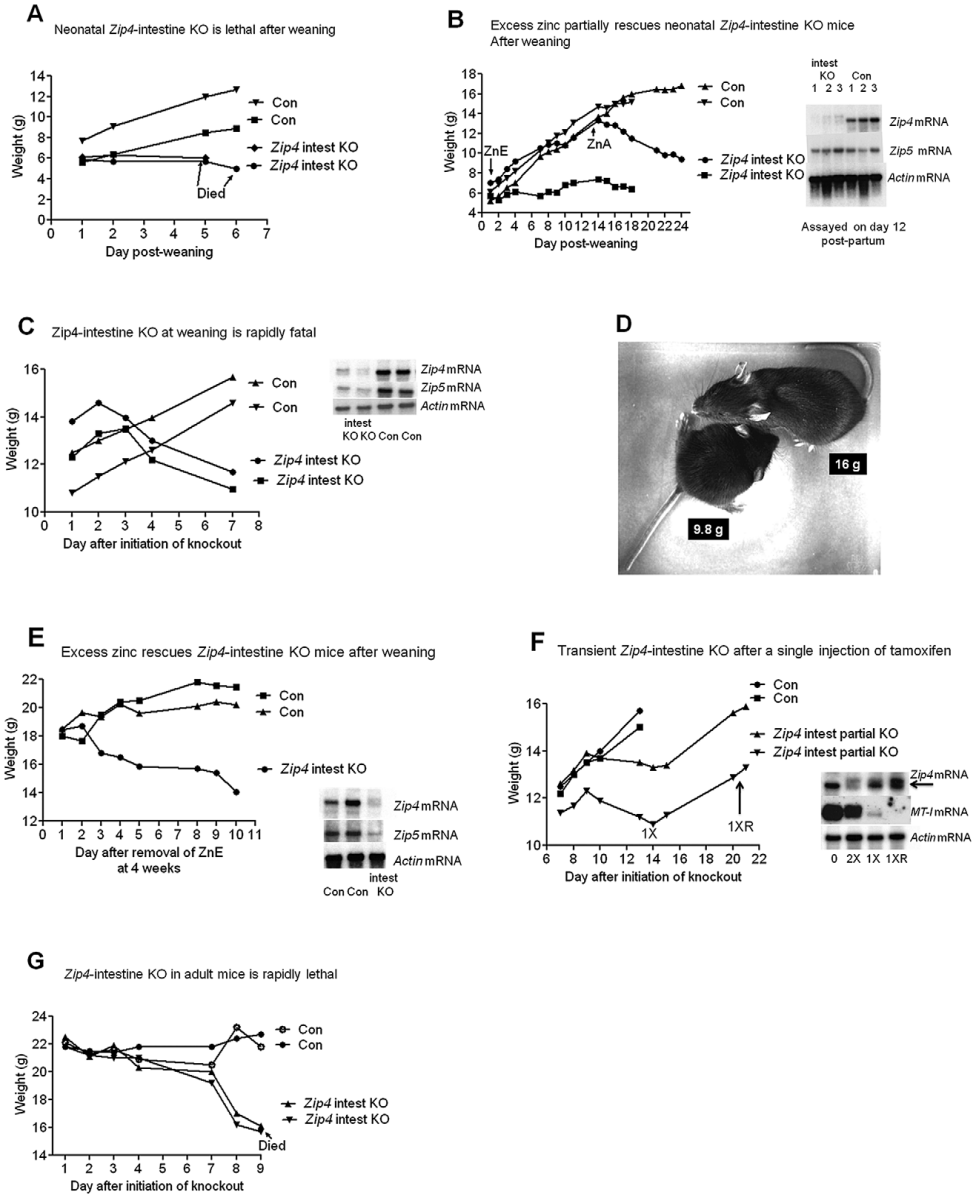
The intestine *Zip4* gene controls growth and viability, but knockout mice can be partially rescued by nursing or excess dietary zinc. (A) Neonatal mice homozygous for the floxed *Zip4* gene and positive for the *vil-CreERT2* gene (*Zip4* intest KO) and littermates homozygous for the floxed *Zip4* gene but negative for the *vil-CreERT2* gene (Con) were injected for 5 consecutive days with tamoxifen beginning 5 days post-partum. After weaning on day 21 their body weights were measured daily (n = 5 to 8 mice per group). These mice were fed normal chow after weaning. Two representative mice from control and *Zip4* intestine knock-out mice are shown but all these mice died by day 7 after weaning (see Figure S1A). (B) *Zip4*-intestine knockout and control neonatal mice were injected with tamoxifen as in A and at weaning these mice (n = 2) were provided access to drinking water containing excess zinc (ZnE). Their body weight was monitored daily for 2 weeks before removing excess zinc and continuing to feed normal chow (zinc adequate feed: ZnA). (Blot Inset) Northern blot hybridization was used to monitor *Zip4*, *Zip5* and *actin* mRNAs in the neonatal intestine on day 12 post-partum, after the 5th injection of tamoxifen (n = 3 mice per group). (C, D, E) Recently weaned mice (*Zip4* intest KO and Con) (n = 5 to 7 per group) were injected for 3 consecutive days or (F) injected once with tamoxifen and their body weight was measured daily. Two representative control and intestine knockout mice are shown. (D) A recently weaned (Con) control mouse (top: 16 g) and a *Zip* intest KO mouse (bottom: 9.8 g) were photographed 8 days after initiation of the knockout. Every *Zip4*-intestine knockout mouse examined died within 16 days of initiation of the knockout. (E) Recently weaned mice (n = 12) were provided excess zinc in the drinking water (ZnE) and normal chow beginning on day 1 after initiation of the knockout. Four weeks later excess zinc was replaced with deionized water and the body weights of the mice were monitored daily. (Blot Inserts) Northern blot hybridization of intestine RNA isolated on day 7 after initiation of the knockout (C) or on day 10 after removal from excess zinc (E). (F) Intestine RNA was isolated from uninjected mice (O), from mice on day 14 after a single injection of tamoxifen (1X) or day 21 (1XR) when the mice had recovered growth (n = 6 to 10 per group). For comparison intestine RNA from *Zip4^FX/FX^:vil-CreERT2* mice injected twice with tamoxifen and harvested on day 4 is shown (2X). (G) Adult *Zip4* intest KO and Control littermates (n = 2 per group; 7 weeks old) were injected for 3 consecutive days with tamoxifen and their body weights were measured daily thereafter. The adult *Zip4*- intestine knockout mice succumbed on day 9 after initiation of the knockout.

Symptoms of AE in humans often appear soon after breast feeding is stopped. Human breast milk provides a rich source of zinc that is easily absorbed by the gut [13]. The effect of knocking out intestine *Zip4* in neonatal mice (5 days post-partum) was therefore examined. Newborn mice were injected with tamoxifen and allowed to suckle until weaning on day 21 post-partum (Figure 2A). At weaning there was no difference in the average body weight of the control (7.93 g: n = 4) and knockout mice (7.96 g: n = 5) and the intestine knockout mice appeared normal. However, the *Zip4*-intestine knockout mice rapidly lost weight after weaning (Figure 2A and Figure S1A) and perished within a week unless provided with excess zinc in the drinking water (Figure 2B). Removal of the excess dietary zinc led to precipitous weight loss and lethality. Northern blot hybridization confirmed that the *Zip4* gene was effectively knocked out in these neonatal mice (Figure 2B, inset). Not all of the recently weaned *Zip4*-intestine knockout mice thrived after providing excess zinc at weaning. Those mice could be rescued from lethality for three weeks post-weaning but failed to gain much body weight during that period (Figure 2B).

The function of intestine *Zip4* in rapidly growing, recently weaned mice was then examined. Recently weaned mice (5 days post-weaning) were injected three consecutive days with tamoxifen and their body weights were measured beginning 1 day after the first injection (Figure 2C and Figure S1B). These mice continue to grow for two to three days after the first injection and then begin to lose weight precipitously. Within a week they lose over 20% of their body weight although they continue to eat a normal amount of food and by d8 they can weigh only 60% as much as their littermate controls (Figure 2D). On day 6 the tibia and quadriceps muscle from the *Zip4*-intestine knockout mice weigh 43 to 50% less than controls while body weight has decreased 23 to 30% relative to littermate controls (n = 4–5; p<0.007). Much of the weight loss involves loss of muscle and bone mass as has been reported to occur during dietary zinc deficiency [14]. Thus, knocking out the intestine *Zip4* gene causes a switch from anabolic to catabolic metabolism in these mice. During the course of these studies we examined many mice and all of the *Zip4*-intestine knockout mice died within two to three weeks. The penetrance of this phenotype is 100% in both male and female mice.

Northern blot hybridization to small intestine RNA demonstrated a loss of *Zip4* mRNA and a dramatic reduction of *Zip5* mRNA (Figure 2C and 2E, insets). ZIP5 is a basolateral zinc transporter expressed predominately in intestinal crypt cells [15]. This mRNA remains unchanged in abundance during severe zinc deficiency in mice [15] but its translation is repressed [12] by a mechanism involving a stem-loop structure in its mRNA [16]. Attenuation of *Zip5* mRNA, whose abundance is not responsive to Zn itself, could suggest injury to the small intestine crypts, as is shown below.

Excess dietary zinc can ameliorate many of the symptoms of AE in humans. Therefore, control and *Zip4*-intestine knockout mice were provided drinking water containing 250 ppm zinc sulfate beginning on the day of the first tamoxifen injection (Figure 2E). A representative animal is shown but 12 mice were analyzed with similar results. Excess zinc prevented the rapid weight loss in the *Zip4*-intestine knockout mice and allowed them to thrive for 4 weeks in this experiment, but withdrawal of the excess zinc led to a rapid loss of weight and lethality within two weeks. The *Zip4* gene remained knocked out for 4 weeks in these mice (Figure 2E, inset) which indicates that it was also knocked out in the intestinal stem cell population, as reported previously using the vil-CreERT2 system [11]. In other experiments it was noted that providing excess zinc could not effectively ameliorate the lethal effects of the *Zip4* knockout if provided later than day 6 (data not shown).

The efficacy of one, two or three consecutive injections of tamoxifen on Cre-ERT2 deletion of the *Zip4* gene was examined using recently weaned mice. As described above, three consecutive injections resulted in effective deletion of the *Zip4* gene even in the intestinal stem cells. In contrast, a single injection of tamoxifen resulted in partial deletion of the *Zip4* gene (Figure 2F, also see Figure S1C). This did not result in lethality; instead those mice grew normally for about a week and then began to slowly lose weight. Thereafter, about half of the mice examined recovered vigorous growth and grew to adult weight (two are shown in Figure 2F) while the other half of these mice continued to slowly lose weight during that time (Figure S1C). The resumption of vigorous growth coincided with active expression of *Zip4* mRNA which had returned to normal levels by three weeks (Figure 2F, inset). These data show that partial loss-of-function of ZIP4 can exert detrimental effects of growth consistent with the findings of potentially hypomorphic alleles of *Zip4* in AE [17] and that heterozygosity of *Zip4* renders mice hypersensitive to zinc deficiency [10].

Many patients with AE require lifelong zinc supplementation. Thus, ZIP4 may function not only during periods of rapid growth but also in full grown adults. Adult mice (7 weeks old) were injected with tamoxifen and then monitored for changes in body weight (Figure 2G). The knockout mice began to lose weight within days of the first injection, lost weight precipitously after 1 week and then succumbed soon thereafter. Thus, the loss of intestine ZIP4 led to a switch from anabolic to catabolic metabolism in adult mice.

### Loss-of-function of the intestine *Zip4* gene has profound tissue-specific effects on the homeostasis of zinc, iron, manganese, and copper

**Figure 3:**
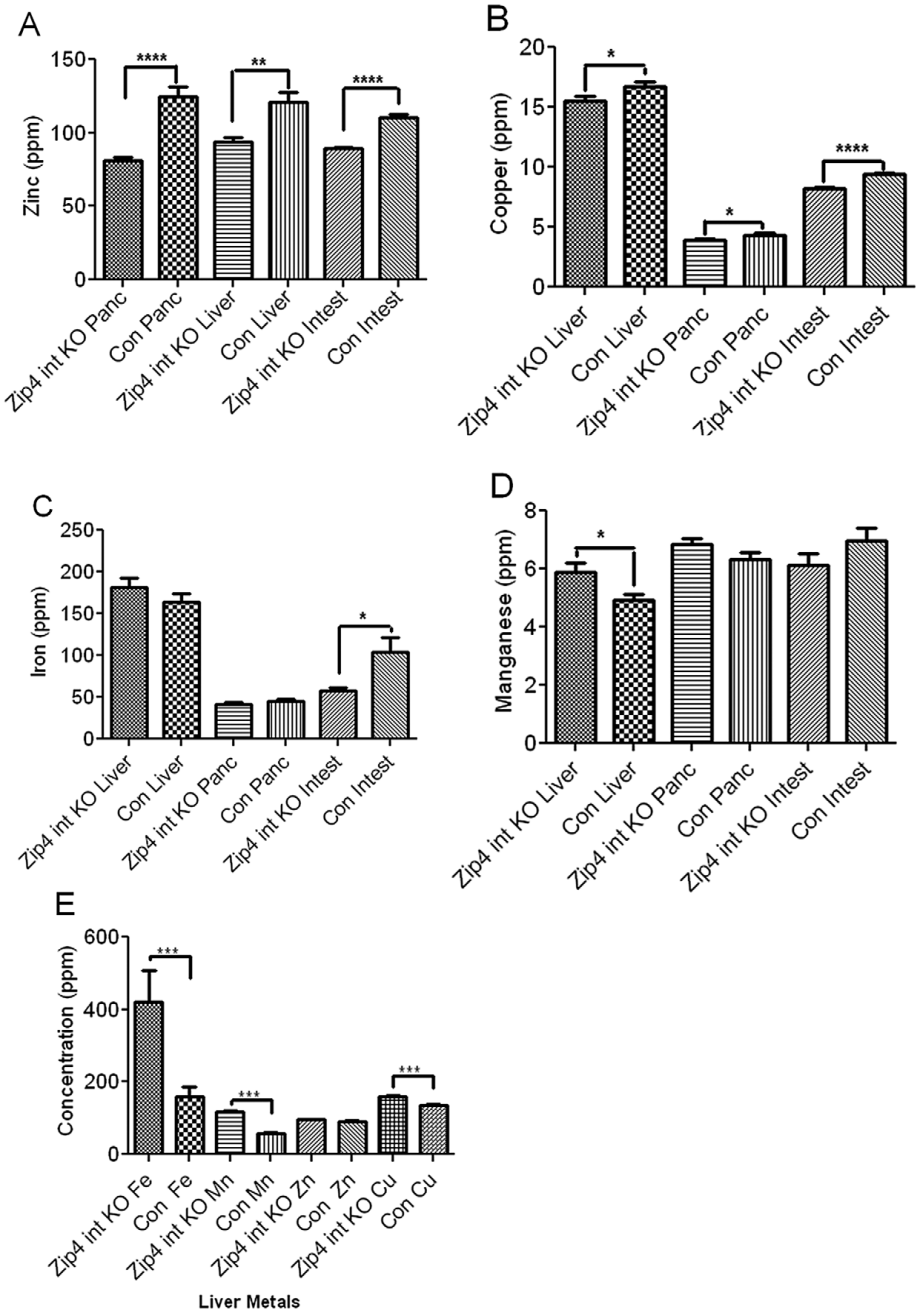
ICP–MS quantification of zinc, iron, manganese, and copper in the small intestine, liver, and pancreas of recently weaned mice after intestine-specific deletion of the *Zip4* gene. Recently weaned mice were injected 3 consecutive days with tamoxifen and the liver (Liver), pancreas (Panc) and small intestine (Intest) from *Zip4*-intestine knockout (intest KO) and control (Con) mice (5 to 10 mice per group) were harvested. Values for zinc (A), copper (B), iron (C) and manganese (D) on day 4 after initiation of the knockout. (E) Values for liver metal concentrations on day 8 after initiation of the knockout. Individual tissue samples were analyzed by ICP-MS for multiple elements including the essential metals. Data are expressed as ppm for each metal ± S.E.M. Statistical significance was determined using the Unpaired T-test (two-tailed). Values were considered different if P<0.05. *indicates P<0.05; *** indicates P<0.001; **** indicates P<0.0001.

ZIP4 is a zinc-specific transporter [18]. Although a recent study suggests that it may also be able to transport small amounts of free copper the physiological relevance of that activity remains to be determined [19]. Herein the effect of knocking out intestine *Zip4* in recently weaned mice, on the homeostasis of several essential metals was examined using ICP-MS. Within 4 days of initiating the knockout, zinc levels in the small intestine, pancreas and liver were significantly reduced (Figure 3A) consistent with a primary function of ZIP4 in the acquisition of dietary zinc. This loss of zinc correlated temporally with the beginning of precipitous body weight loss after the knockout. Although, by day 4 a small but statistically significant loss of copper (Figure 3B) also occurred in these organs, the largest copper loss was in the small intestine which also lost ∼50% of its iron (Figure 3C). Thus, the loss of ZIP4 in the intestine led to large reductions in small intestine zinc, iron and copper content suggesting possible injury to the intestine, as was found to be the case. An increase in manganese was noted in the liver (Figure 3D), but no other significant changes in other elements in these tissues were found on day 4. Remarkably, by day 8 after initiation of the knockout, iron and manganese were twice as high in the liver of Zip4-intestine knockout mice versus control and hepatic copper had also increased significantly (Figure 3E). In contrast, zinc levels were normalized in the liver which suggests that zinc had been redistributed as body weight declined. The accumulation of iron in the liver during zinc deficiency has been reported previously [20], [21]. Taken together, these results show that intestine ZIP4 is essential for the homeostasis of several essential metals.

### Loss-of-function of the intestine *Zip4* gene rapidly alters gene expression in the small intestine

**Figure 4:**
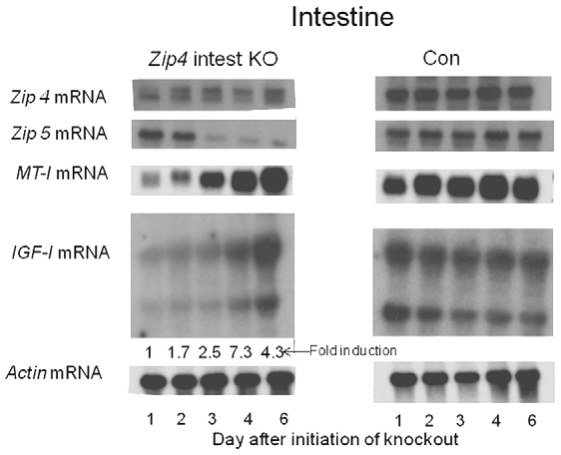
Northern blot analysis of temporal changes in small intestine gene expression after intestine-specific deletion of *Zip4*. Recently weaned mice were injected 3 consecutive days with tamoxifen and the small intestines from *Zip4*-intestine knockout (*Zip4* intest KO) and control (Con) mice (3 mice per group) were harvested on the indicated days after initiation of the knockout and analyzed for *Zip4*, *Zip5*, *IGF-1* and *actin* mRNAs by Northern blotting. *IGF-1* has multiple transcripts and the control blot is over-exposed relative to the *Zip4*-intestine blot. *IGF-1* transcripts in the *Zip4*-intestine knockout intestine were also quantified by qPCR using primers which amplify the active IGF-1 peptide encoding region of all *IGF-1* transcripts. The fold change in *IGF-1* mRNA relative to day 1 is presented below the autoradiograph.

Being able to temporally control the intestine knockout of *Zip4* enables us for the first time to monitor the rapid effects of loss-of-function of a zinc transporter in an animal. This model of AE compresses the time frame from months in humans to a few days in mice. This allows for examining primary versus secondary effects of losing *Zip4* function. To that end Northern blot hybridization and quantitative PCR were used to monitor changes in *Zip4*, *Zip5* and *metallothionein- I* (*MT-I*) mRNAs, zinc homeostatic genes, in the small intestine (Figure 4). In addition changes in *IGF-1* mRNA, a potent mitogen in the intestine [22], were monitored.

A single injection of tamoxifen resulted in a large loss of native *Zip4* mRNA in the small intestine 24 hr later (Figure 4). By one day after the second injection (day 2), little or no native *Zip4* mRNA was detectable. *Zip5* mRNA abundance dropped precipitously by day 3 while MT-I mRNA was reduced in abundance within a day of the first tamoxifen injection but increased again by d4 and remained high (Figure 4). Thus, deletion of the intestine *Zip4* gene occurs rapidly in this model and leads to significant changes in the abundance of several mRNAs involved in zinc homeostasis. The loss of *Zip5* expression suggests that injury to the intestine may occur rapidly after deletion of the *Zip4* gene, as *Zip5* mRNA abundance is not zinc-responsive.

Deletion of the intestine *Zip4* gene led to a large increase in the abundance of *IGF-I* mRNA. Several *IGF-I* transcripts are present in the intestine. Quantitative PCR of the region encoding IGF-I in all of the reported transcripts confirmed this Northern blot finding and established that this mRNA is induced up to 7-fold within a few days (Figure 4). There were no changes in the abundance of *IGF-I* transcripts in control littermates (Figure 4, Con). The Con blot was over-exposed relative to that of the knockout mice.

### Loss-of-function of the intestine *Zip4* gene leads to a rapid loss of Paneth cell zinc, repression of Sox9, and induction of mucin in Paneth cells, which is accompanied by disorganization of crypt and villus morphology

**Figure 5:**
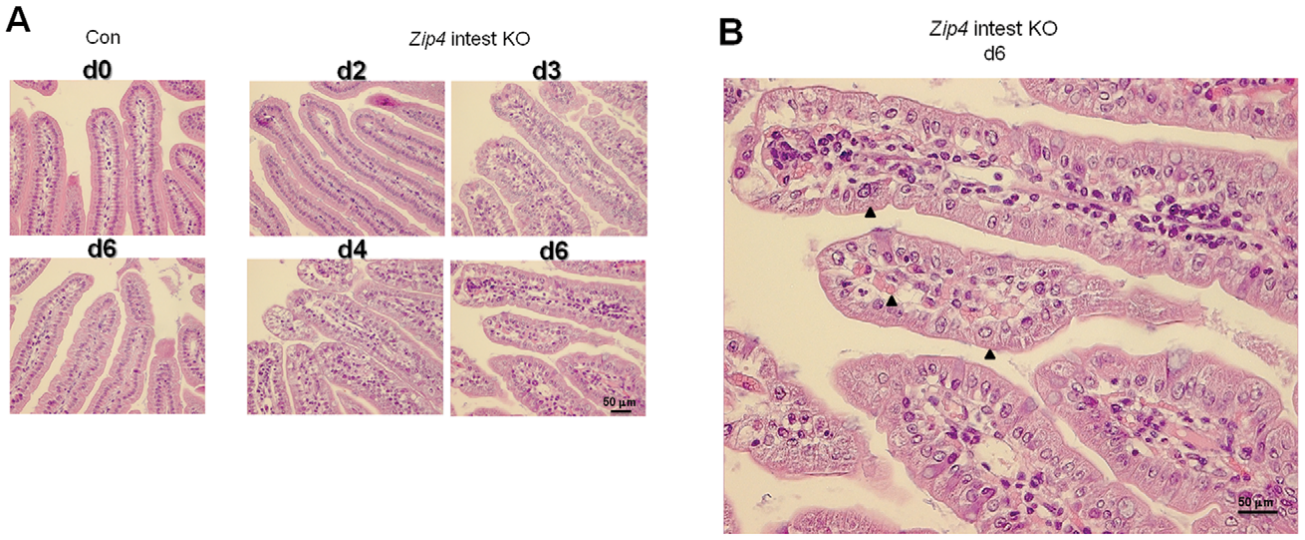
Progressive disruption of villus integrity after intestine-specific deletion of *Zip4*. Recently weaned mice were injected 3 consecutive days with tamoxifen and the small intestines from control (Con) and *Zip4*-intestine knockout (*Zip4* intest KO) mice (5 mice per group) were harvested on the indicated days after initiation of the knockout. (A) Sections of small intestine were stained with hematoxylin-eosin. Sections from control day 0 and day 6 intestine are shown (2 left side panels), whereas sections from Zip4-intestine knockout days 2–4 and day 6 intestine are shown (4 right side panels). (B) A blow-up view of the *Zip*-intestine knockout d6. Black arrowheads demarcate examples of cubodial epithelial cells and RBCs in the lamina propria.

**Figure 6:**
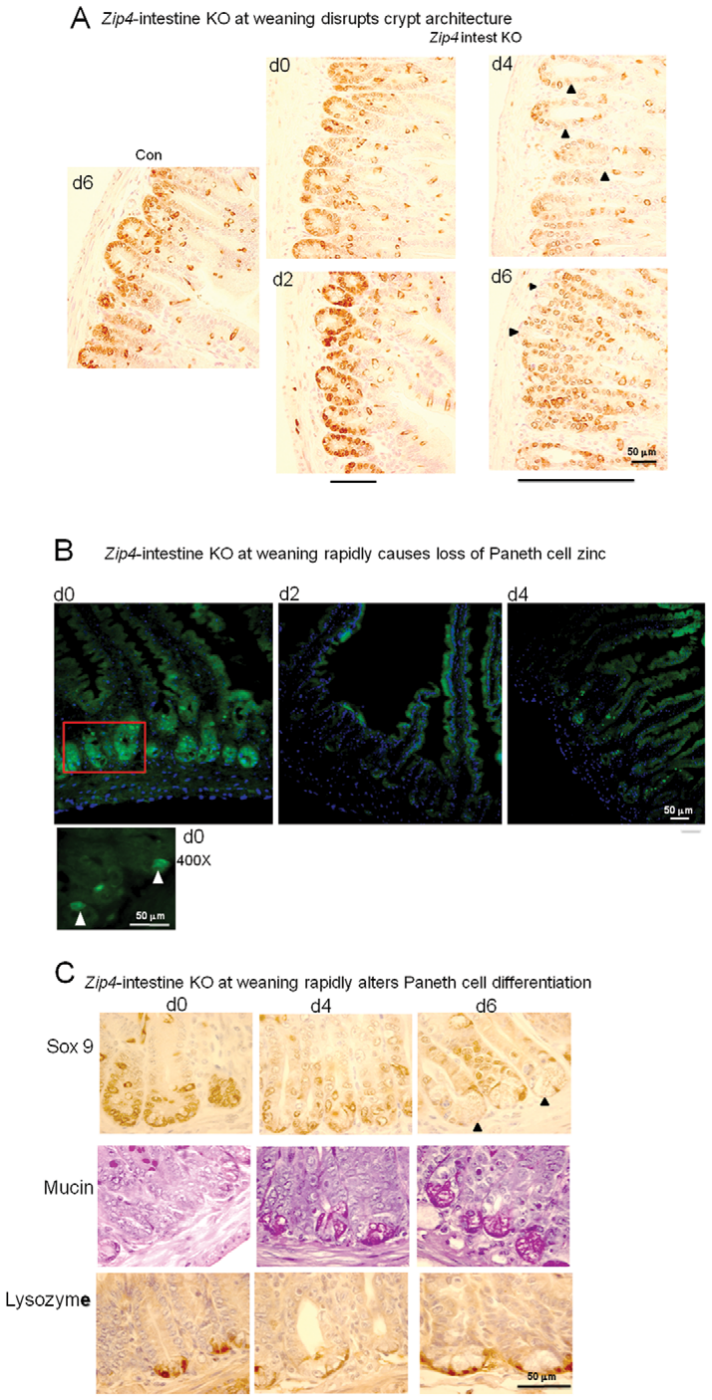
Intestine-specific deletion of *Zip4* leads to progressive disruption of crypt architecture, the rapid loss of zinc, and reprogramming of Paneth cells. Recently weaned mice were injected 3 consecutive days with tamoxifen and the small intestines from *Zip4*-intestine knockout (*Zip4* intest KO) and control (Con) mice (5 mice per group) were harvested on the indicated days after initiation of the knockout. (A) Paraffin sections of small intestine were processed for IHC using an antibody against mouse Sox9. Brown color indicates positive immunostaining. Bars below the d2 and d6 panels of *Zip4* -intestine knockout section demarcate the approximate length of the crypts in each panel and black arrowheads indicate Sox9 negative cells interspersed among the positive cells (d2) and Sox9 negative cells at the base of the crypts (d6). (B) Frozen sections of small intestine from *Zip*4-intestine knockout mice were incubated with ZP1 and photographed using the GFP cube. Brightness of the sections was reduced equally in Photoshop. Green indicates positive signal for zinc-induced ZP1 fluorescence. A higher power view of the red boxed region in the d0 panel is shown below and white arrows demarcate labeled Paneth cells. (C) Paraffin sections were processed for IHC using an antibody against mouse Sox9 or lysozyme. Brown color indicates positive immunostaining. Serial sections were also stained with PAS to detected mucin. Dark pink to red color indicates positive PAS staining. Black arrows demarcate Paneth cells in the d6 Sox9 stained panel. Tamoxifen injections did not affect Sox9, lysozyme or mucin staining of small intestine sections from control littermates (not shown).

**Figure 7:**
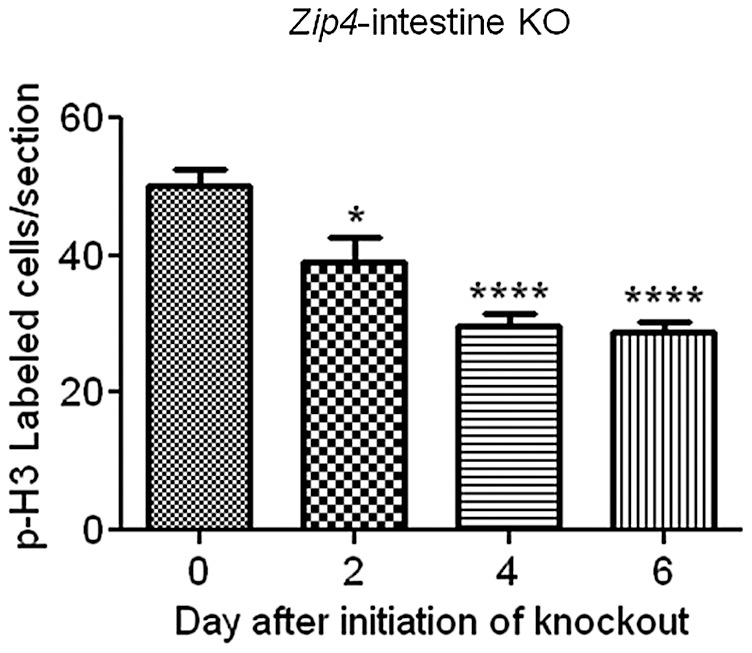
Quantification of phosphorylated-histone H3 labeled cells in sections of small intestine after intestine-specific deletion of *Zip4*. Recently weaned mice were injected 3 consecutive days with tamoxifen and the small intestines from *Zip4*-intestine knockout mice (5 mice per group) were harvested on the indicated day after initiation of the knockout. Paraffin sections were processed for IHC using an antibody against Ser 10 phosphorylated-histone H3. The number of positive cells per field of view was counted in multiple sections from several mice and the values shown are the mean ± S.E.M. Statistical significance was determined using the Unpaired T-test (two-tailed). Values were considered different if P<0.05. *indicates P<0.05; **** indicates P<0.0001. Tamoxifen injections did not affect phospho-H3 labeling in small intestine sections from control littermates (not shown).

Histological examination of the small intestine after *Zip4* knockout revealed progressive and profound disorganization of villus (Figure 5) and crypt architecture (Figure 6) and significantly diminished cell division within days of the knockout (Figure 7). Villus histology was apparently normal on day 2 after initiation of the knockout but became progressively disorganized over the next 4 days (Figure 5A). By day 6 the epithelial cells had lost much of their columnar morphology with basal nuclei and had become cuboidal with centric nuclei (Figure 5B). The lamina propria appeared disorganized, occupied substantially more space in the villi and often contained red blood cells and cells with pyknotic nuclei.

Crypts were examined by localizing the transcription factor Sox9 in sections of small intestine. Sox9 (sex determining region Y- box9) marks the stem/progenitor cell population in the crypts [23] as well as the villus enteroendocrine cells [24] and it regulates cell proliferation and Paneth cell differentiation [25]. In control mice and *Zip4*-intestine knockout mice before tamoxifen injections and on day 2 after initiation of the knockout, Sox9 was localized to the crypts (Figure 6A) and to dispersed enteroendocrine cells in the villi (not shown), as reported previously [24]. However, after day 2 the architecture of the crypts progressively became disorganized and elongated, and Sox9 positive cells were increasingly detected extending up into the villus epithelium with Sox9 negative cells interspersed among the positive cells (Figure 6A). By days 4 and 6, Sox9 negative cells were found at the base of many crypts (Figure 6A and 6C). The location of these cells at the base of the crypts suggested that these were Paneth cells; however, these cells contained large cytoplasmic vacuoles not found in normal Paneth cells.

Paneth cells contain large amounts of labile zinc which can be detected by staining with the zinc-specific fluorophore ZP1 [26]. ZP1 staining was readily detectable in the intestinal crypts before initiation of the *Zip4* knockout (Figure 6B). When viewed under high power ZP1 staining was restricted to one or two cells at the base of the crypts (Figure 6B; 400X panel) whereas under low power the fluorescence signal was dispersed throughout the crypt. ZP1 staining was lost by day 2 after initiation of the knockout and remained undetectable in the Paneth cells on day 4. Thus, zinc is rapidly depleted in Paneth cells when ZIP4 function is lost.

The antibacterial enzyme lysozyme is abundantly produced by Paneth cells [27]. IHC localization of lysozyme in intestine sections from control and *Zip4*-intestine knockout mice confirmed that Paneth cells accumulate large vacuoles, and have reduced Sox9 and zinc when *Zip4* is knocked out (Figure 6C). Paneth cells, enteroendocrine and goblet cells are thought to arise from a common precursor [28]. Paneth cells constitute the niche for Lgr5-positive columnar stem cells in the crypt base [29], [30]. The Lgr5-positive columnar cells can give rise to all epithelial lineages [29]. PAS staining of mucins revealed normal goblet cells interspersed in the villus epithelium of uninjected and knockout mice (Figure S2). PAS staining of the small intestine from *Zip4*-intestine knockout mice demonstrated positive staining of the large vacuoles in Paneth cells by day 4 (Figure 6C). This staining was resistant to α-amylase and was therefore not due to glycogen (data not shown). Enteroendocrine (Sox9+) cells and goblet (PAS+) cells are long-lived in the villus epithelium and appeared to be normal in numbers after knocking out intestine *Zip4*, at least within the time period examined (Figure S2). No change in Paneth cells were seen in control littermates (Con) that had been similarly injected with tamoxifen and processed in parallel (data not shown). These results indicate that loss-of-function of intestine ZIP4 leads to a rapid loss of Paneth cell zinc which is followed by changes in Paneth cell morphology and phenotypic reprogramming.

### Loss-of-function of the intestine *Zip4* gene leads to repressed cell division

The relative amount of cell division in the small intestine was determined since Sox9 plays a role in intestinal stem cell proliferation and the crypts are critical for renewal of the epithelium. Cell division was monitored by IHC for phosphorylated (Ser 10) histone H3 and quantified by counting positively stained cells in multiple fields of view (5 or more) in sections from several (4 or 5 mice) control and *Zip4*-intestine knockout mice (Figure 7). On day 2 after initiation of the knockout, cell division was measurably repressed (22%) in the small intestine and by day 4, cell division was reduced by 47% and remained repressed on day 6. No reduction in cell division after tamoxifen injections was noted in control littermates (data not shown). These results reveal that intestine ZIP4 functions to maintain the structural and functional integrity of the small intestine.

### Loss-of-function of the intestine *Zip4* gene causes a rapid loss of phosphorylated S6 ribosomal protein in the villus epithelium followed by increased phosphorylated S6 ribosomal protein in the crypts

**Figure 8:**
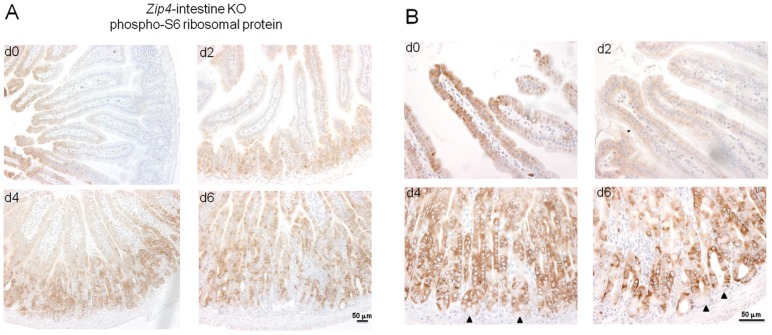
IHC detection of phosphorylated S6 ribosomal protein, as a read out for mTOR1 activity, reveals a loss of villus staining followed by increased staining of crypts after intestine-specific deletion of *Zip4*. Recently weaned mice were injected 3 consecutive days with tamoxifen and the small intestines from *Zip4*-intestine knockout mice (5 per group) were harvested on the indicated days after initiation of the knockout. (A) Paraffin sections were processed for IHC using an antibody against mouse phospho-S6 ribosomal protein. Brown color indicates positive immunostaining. (B) High power views of panels in (A). Black arrows demarcate Paneth cells. Tamoxifen injections did not affect phospho-S6 ribosomal protein staining in sections of small intestine from control littermates (not shown).

Given the repressive effects of loss-of-function of ZIP4 on small intestine cell division and structural integrity leading to a switch from anabolic to catabolic metabolism in mice, we examined the effects of this knockout on the activity of mammalian target of rapamycin (mTOR1) in the intestine. mTOR1 plays an important role in growth regulation and intestinal cell migration by integrating signals from nutrients, energy status, growth factors and stresses thereby controlling protein translation [31]–[33]. Ribosomal protein S6 kinase is activated by mTOR1 leading to phosphorylation of ribosomal protein S6 and enhanced translation [31]. Therefore, the status of phosphorylated ribosomal protein S6 was monitored by IHC in the small intestine of control and *Zip4*-intestine knockout mice (Figure 8). Phosphorylated ribosomal protein S6 was readily detectable in the enterocytes of the villus tips in control mice. Little or no staining was detected in crypts (Figure 8A). However, by d2 after initiating the knockout staining in the crypts became apparent while that in the villus tips was diminished (Figure 8B), and by d4 crypt staining was strong and villus staining was weak or absent. On day 6 crypt staining remained strong and cells interspersed in the villus epithelium stained positive for phosphorylated ribosomal S6 protein. Closer examination of the stained crypts revealed an absence of staining in the Paneth cells (Figure 8B). These results indicate that loss of ZIP4 function in enterocytes leads to a rapid and prolonged diminution in mTOR1 activity and impaired protein synthesis in those cells. Remarkably this is accompanied by enhanced mTOR1 activity in the stem cell niche, but not in Paneth cells.

### Loss-of-function of the intestine *Zip4* gene causes rapid and transient changes in energy metabolism in the liver

**Figure 9:**
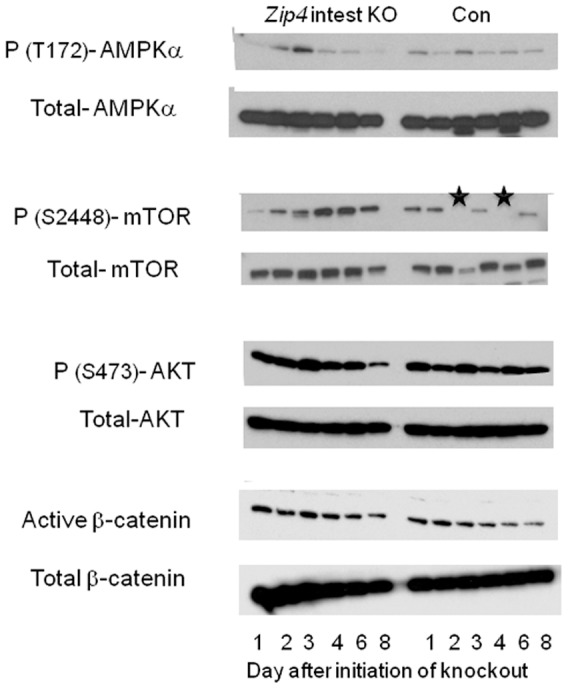
Western blot analysis of phosphorylated and total AMPKα, mTOR1, AKT, and β-catenin in the liver after intestine-specific deletion of *Zip4*. Recently weaned mice homozygous for the floxed *Zip4* gene and positive for the *vil-CreERT2* gene (*Zip4* intest KO) and littermates homozygous for the floxed *Zip4* gene but negative for the *vil-CreERT2* gene (Con) were injected for 3 consecutive days with tamoxifen. Livers were harvested on the indicated day after initiation of the knockout and pooled from 3 to 5 mice per group. Soluble proteins were analyzed by Western blotting using the indicated antibodies. Black stars in the WT p-(S2448)- mTOR blot indicate samples that were partially degraded. mTOR1 is a 289 kDa protein.

Unlike the intestine, the liver in *Zip4* intestine knockout mice apparently does not undergo obvious morphological changes and the liver to body weight ratio remains relatively constant during the first week or so after initiating the knockout (data not shown). In contrast there is a rapid loss of bone and muscle mass and body weight in these mice. The liver plays a critical role in metabolism; therefore, the status of AMP-activated protein kinase (AMPK) and mTOR1 activity in the liver were examined by Western blotting (Figure 9). The activity of AMPK is allosterically increased by AMP binding which leads to phosphorylation and activation of AMPK. When ATP generation is attenuated, AMPK becomes activated and phosphorylates many substrates leading to increased activation of catabolic pathways and reduced activation of anabolic pathways [32]. As mentioned above, mTOR1 activity enhances anabolic metabolism by supporting translation and transcription.

Western blot analysis of liver proteins revealed that phosphorylated (T172)-AMPK increased soon after the knockout of intestine *Zip4* reaching a peak on day 3 before rapidly declining (Figure 9). This decline was accompanied by increased and prolonged phosphorylation (S2448) of mTOR1. In contrast, little change was noted in the phosphorylation of AKT (S473) or active β-catenin until day 8. Taken together, these results show that the loss-of-function of intestine ZIP4 rapidly causes a switch from anabolic to catabolic metabolism in the liver reflecting an increased AMP/ATP ratio. However, this effect was transient and mTOR1 activity was restored when the mice began wasting muscle and bone which would provide amino acids and other energy sources for liver metabolism. Thus, unlike the small intestine (as well as bone and muscle) the liver is only transiently affected by the loss-of-function of intestine ZIP4.

## Discussion

Herein we describe the creation of a mouse model of the rare pseudo-dominant human genetic disorder of zinc metabolism AE. Being able to control the timing and extent of the knockout of enterocyte *Zip4* in mice, and the rapid progression of the disease thereafter, allowed us for the first time to effectively address underlying mechanisms of AE. A central remaining question in our understanding of AE has been to define the role of intestinal *Zip4* in the etiology of this disease. Mouse *Zip4* expression can be detected in several cell-types [18], [34]. Human *Zip4* expression is also detected in several organs (NCBI Unigene EST profile) and fibroblasts from AE patients show diminished zinc uptake and zinc content leading to the suggestion that loss-of-function of ZIP4 in organs other than the intestine could contribute to the pathology of AE [35]. Our data demonstrate that the most profound phenotypes of AE, severe growth retardation and morbidity, reflect a loss-of-function of ZIP4 only in intestinal enterocytes, that was predicted from studies of patients with AE nearly 30 years ago [2]. A significant difference between this mouse model and human AE is the lack of severe dermatitis and alopecia in mice. This likely reflects the fact that *Zip4* is actively expressed in human skin (NCBI Unigene EST profile) whereas it is not actively expressed in mouse skin [18].

In mice, growth retardation and/or weight loss occur rapidly after enterocyte *Zip4* is knocked out and the disease progresses to morbidity within a few days accompanied by a switch from anabolic to catabolic metabolism, unless mice were nursing or given access to high zinc concentrations in their water. Similarly, humans with AE respond favorably to increased dietary intake of zinc. How zinc is absorbed from the gut in the absence of ZIP4 is not known. However, uptake of zinc by L-type calcium channels has been reported [36] and several other Zip and ZnT proteins are expressed in the intestine and could contribute to zinc uptake when zinc levels are very high [37]–[39].

Our studies revealed that one of the most rapid effects of loss-of-function of intestine ZIP4 is the loss of labile zinc from Paneth cells. This may therefore be an initial event in the progression of AE. Studies of patients with AE, also reported nearly 35 years ago, revealed changes in Paneth cells including the accumulation of abnormal inclusion bodies and giant granules, which were reversed when patients were given oral zinc [40]–[42]. Zinc deficiency or prolonged fasting has been shown to cause the loss of Paneth cell zinc, as measured by reactivity with the zinc chelator dithizone [43] and dithizone injection causes selective killing of Paneth cells [44]. However, zinc deficiency alone in rats does not lead to abnormal Paneth cell morphology [45] which suggests that factors in addition to zinc loss may contribute to the pathology of Paneth cells in AE patients.

The loss-of-function of intestine ZIP4 rapidly alters the phenotype of Paneth cells which are long-lived, suggesting that preexisting Paneth cells undergo reprogramming. The rapid loss of zinc in these cells is followed by reduction of Sox9 and the accumulation of mucin positive Paneth cells. Sox9 regulates cell proliferation in the small intestine epithelium as well as Paneth cell differentiation and repression of mucin (Muc2) expression [23], [46]. Sox9 is regulated by the Wnt pathway [23] which suggests that ZIP4 may play a role in regulating signal transduction in Paneth cells via the Wnt-APC pathway. A role of zinc in controlling glycogen-synthase kinase-3β (GSK-3β) activity and, by extension the Wnt-APC pathway, has been reported [47]–[49]. Increased zinc leads to increased phosphorylation of GSK-3β, the accumulation of β-catenin and increased signal transduction. Therefore, our data are consistent with the concept that the loss of zinc in Paneth cells leads to reduced phosphorylation of GSK-3β, increased turn-over of β-catenin, reduced Sox9 expression, and reprogramming of crypt Paneth cells.

Loss-of-function of ZIP4 also resulted in a reduction in cell division in the crypts which may be a consequence of the altered Paneth cell phenotype and crypt dysplasia. A recent report revealed that Paneth cells contribute to the intestinal stem cell niches by expressing several growth factors, most importantly Wnt, which support those stem cells [30]. Perturbed function of Paneth cells would negatively affect Lgr5 columnar stem cells which are interspersed between Paneth cells in the crypts [29], [30]. These stem cells are able to give rise to all the epithelial lineages *in vivo*, can generate crypt like structures *in vitro*, and there is a strong correlation between the number of Paneth cells and the number of Lgr5 stem cells in the intestine *in vivo* [29], [30]. Therefore, impaired Paneth cell function contributes to reduced cell division in the small intestine through its effects on the stem cell niche.

It is interesting to note that *Zip4* expression in the mouse intestine is restricted to villus enterocytes and this protein is not detected by IHC in the crypts [15], [18]. Although this cannot exclude the possibility that weak expression of *Zip4 i*n the crypts may contribute to the changes noted in Paneth cells after this gene is knocked out, a more plausible explanation is that loss-of-function of villus enterocyte ZIP4 leads to loss of Paneth cell zinc and it is this loss of zinc which initiates the reprogramming of Paneth cells rather than a loss of a direct signal transduction function of ZIP4 in Paneth cells. The blood circulation provides an intimate connection between the villus and crypt such that enterocyte absorbed nutrients, including Zn, are available at high concentrations to cells in the crypt.

There is evidence for zinc transporters affecting cell signaling. Studies of knockout mice suggest that Slc39a14 (ZIP 14) represses phosphodiesterase activity leading to enhanced systemic growth, but the loss-of-function of ZIP14 is not lethal and has only modest effects on growth [50], unlike the loss of ZIP4 function. Similarly Slc39a13 (ZIP 13) has been shown to play a role in connective tissue development where it affects BMP/TGF-β signaling [51]. The preponderance of data suggests that zinc mediates the effects of these zinc transporters [9]. However, ZnT1 has been shown to directly interact with EVER proteins [52] leading to altered intracellular distribution of zinc, and ZnT1 can interact with Raf-1 kinase through its c-terminal domain leading to activation of ERK signaling [53]. No binding partners of ZIP4 have been reported.

In contrast to the pattern of *Zip4* expression in villi, *Zip5* expression is most active in crypt cells where it localizes to the basolateral membrane when dietary zinc is adequate [15]. This protein is normally degraded during periods of zinc deficiency but the mRNA stays associated with polysomes [12] and its translation is regulated by a conserved stem-loop structure in the 5′-untranslated region and two microRNAs in response to zinc [16]. The reduction of *Zip5* mRNA abundance after knocking out the enterocyte *Zip4* gene occurs concomitantly with crypt dysplasia. However, intestine-specific knockout of mouse Zip5 is asymptomatic (Geiser and Andrews, unpublished results) which suggests that loss of *Zip5* expression in the crypts of the *Zip4*-intestine knockout mice reflects a loss of the differentiated state of intestinal crypt cells.

Another rapid effect of loss-of-function of intestine ZIP4 is the attenuation of mTOR1 activity, as measured by phosphorylation of S6 ribosomal protein, in the villus epithelium. This indicates that protein synthesis in villus enterocytes is rapidly diminished after ZIP4 is lost [31]. The intestine has a high rate of protein synthesis to supply digestive enzymes and transporters necessary for enterocyte nutritional functions, and total intestine utilization of amino acids can account for half of whole-body utilization [54]. Attenuation of protein synthesis in the intestine impairs growth via malnutrition. For example, the loss-of-function of intestine Slc6a19, the neutral amino acid transporter, impairs growth and body weight control in mice and reduces mTOR1 activity in the intestine [55] but is not lethal. Whether loss-of-function of ZIP4 attenuates amino acid uptake by the small intestine remains to be determined but amino acids and growth factors play essential roles in mTOR1 signaling [31] and amino acid stimulation of mTOR1 signaling and protein synthesis are required for intestinal cell migration [33]. The attenuation of mTOR1 activity in villus enterocytes likely contributes to the disorganization of the epithelium which occurs after *Zip4* is knocked out. Dietary zinc enhances mTOR1 signaling and GSK-3β phosphorylation in skeletal muscle and liver leading to increased protein synthesis [49]. Similar to Paneth cells, villous enterocytes show altered morphology when intestine *Zip4* is knocked out. Combined with the reduced rate of cell division and reduced mTOR1 activity, the loss of normal columnar morphology of enterocytes is consistent with a loss of differentiated function, which likely is a major contributor to growth failure in these mice.

Our studies revealed that loss-of-function of intestine ZIP4 leads to transient activation of hepatic AMPK, a cellular energy sensor which is activated when AMP levels increase [32] and which antagonizes mTOR1 activity [32]. Loss of zinc likely leads to attenuated mTOR1 signaling in the liver through reduced production of ATP and activation of AMPK. This is followed by repletion of hepatic zinc due to the redistribution of zinc, and other nutrients, from bone and muscle stores as the knockout mice begin to lose body mass. This leads to attenuation of AMPK activity and increased mTOR1 activity. By extension, the attenuation of villous mTOR1 activity in response to the loss-of-function of ZIP4 in villous enterocytes likely reflects a response to diminished zinc uptake rather than a signaling function of the ZIP4 protein in enterocytes.

In addition to the potential reduction in intestinal uptake of amino acids and zinc which can control mTOR1 activity, it is also possible that loss-of-function of ZIP4 attenuates IGF-1 signaling in the intestine. Intestine IGF-1 mRNA is induced after *Zip4* is knocked out and this growth factor is a potent tropic mitogen in the intestine [22]. IGF-1 mRNA is induced in intestinal subepithelial myofibroblasts in response to stimulation by glucagon-like-peptide 2 secreted from intestinal L-cells, and IGF-1 enhances crypt cell proliferation by stimulating β-catenin signaling in non-Paneth cells [22]. The finding that IGF-1 is induced but mTOR1 activity is repressed in the villus epithelium suggests that IGF-1 signaling is attenuated when intestine ZIP4 is lost. Intracellular zinc concentrations have been shown to modulate IGF-1 signaling by affecting protein tyrosine phosphatase activity [56]. High zinc is inhibitory leading to enhanced IGF-1 signaling whereas zinc deficiency impairs IGF-1 signaling [57].

Loss of mTOR1 activity in the villus epithelium occurred soon after *Zip4* was knocked out in the intestine. In contrast, the activity of mTOR1 was increased in crypts thereafter but this failed to lead to enhanced cell proliferation. The activation of mTOR1 in crypts may reflect a stress response or a compensatory response to crypt dysplasia in the intestine [32]. Increased IGF-1 signaling may lead to activation of mTOR1 in crypt cells causing enhanced translation but fails to enhance cell division due to impaired β-catenin signaling. Clearly a complicated interplay between loss of zinc and altered signal transduction cascades occurs in the intestine leading to diminished integrity of the small intestine in AE.

Loss-of-function of intestine ZIP4 led to altered homeostasis of several essential metals over time. Although zinc was rapidly lost from intestine, pancreas and liver soon after the *Zip4* knockout, normal levels of zinc re-accumulated in the liver as body weight, bone and muscle mass were lost. In contrast, iron and manganese accumulated in the liver. Accumulation of iron in the liver during zinc deficiency has been previously reported but manganese accumulation has not. Zinc deficiency can result in alterations in iron transporter, storage, and regulatory proteins which facilitate iron accumulation [20]. In addition ZIP14 (Slc39a14) has been suggested to play a role in the accumulation of non-transferrin bound iron in the liver [58]. It is possible that reduced expression of ATP13A2, which has been shown to reduce cellular manganese concentrations, occurs in response to severe zinc deficiency in our model [59], and a recent study revealed that ZnT10 (Slc30a10) plays a key role in manganese transport in humans [60], [61]. Whether enhanced expression or activity of ZIP14 and/or repression of ZnT10 play roles in the accumulation of iron and manganese, respectively, in the liver in response to loss-of-function of intestine ZIP4 remains to be determined. Accumulation of very high levels of iron in the liver (20 to 100×) can lead to hemochromatosis and can inhibit manganese uptake by mitochondria leading to mitochondrial dysfunction [62]. Therefore, the slow accumulation of hepatic iron and manganese in the *Zip4*-intestine knockout mice could eventually contribute to liver injury if very high levels are accumulated later during the disease process. Perhaps humans with AE may be at risk for hemochromatosis at advanced stages of the disease.

In summary, our studies report the first conditional knockout of a member of the mammalian ZIP family of zinc transporters. By controlling the timing and extent of the knockout of the intestine *Zip4* gene in mice we were able for the first time to monitor temporal and tissue-specific effects of the loss-of-function of ZIP4 and provide insights into the etiology of AE. The results suggest that Paneth cell dysfunction leading to disruption of the stem cell niche and loss of villus enterocyte integrity are primary causes of AE. These changes likely reflect diminished zinc uptake by ZIP4 and are followed by a switch from anabolic to catabolic metabolism in the mouse leading to dramatic weight loss, dyshomeostasis of several essential metals and ultimately lethality in the absence of excess dietary zinc.

## Materials and Methods

### Animals

Experiments involving mice were performed in accordance with the guidelines from the National Institutes of Health for the care and use of animals and were approved by the Institutional Animal Care and Use Committee. Mice were maintained on normal mouse chow (zinc adequate:ZnA) and where indicated were also provided with drinking water containing 250 ppm ZnSO_4_ (zinc excess: ZnE).

### Recombineering of a floxed Zip4 (Slc39a4) gene

We previously described the structure of the mouse *Zip4* (Slc39a4) gene in detail [15], [18]. The final structure of the *floxed Zip4* gene targeting vector created herein and of the targeted chromosomal locus is shown in Figure 1. BAC recombineering [63] using galK selection was employed to manipulate the *Zip4* gene [64] A 9180 bp region containing the *Zip4* gene plus 2.3 kb of 5′-flanking DNA and 3.2 kb of 3′ flanking DNA was captured from BAC ct7-43303 and gap-repaired in a conditionally amplifiable [65] BAC-based vector (P[acman]-M-KO) [66] that allows for negative selection (*HSV-TK*) in ES cells and positive selection in bacteria (*ampicillin*). A *LoxP* site engineered to contain an *Xho I* restriction site was inserted into a poorly conserved region in intron 5 and a second *LoxP* site was inserted immediately downstream of exon 12, the last exon in this gene. An *mc1-neomycin* cassette was inserted about 250 bp downstream of exon 12. The targeting vector's structure was confirmed for the entire gene including the neomycin cassette by DNA sequencing and the ends of the captured regions were sequenced to verify that no rearrangements or mutations had occurred. In addition, the functionality of the *LoxP* sites was confirmed by transformation of the final targeting vector into bacteria (EL 350) that express Cre recombinase under control of an arabinose inducible promoter [63].

### Targeted insertion of the floxed Zip4 (Slc39a4) gene in embryonic stem cells

The Transgenic and Gene-Targeting Institutional Facility at the KU Medical Center generated targeted ES cell clones and performed blastocyst injections. The *Zip4* ^FX^ targeting vector was linearized with *Not1* and electroporated into E14 embryonic stem (ES) cells. Selected colonies were screened by long range PCR using LA-Taq (TaKaRa Bio, Inc.) and primers which flank the *Zip4* region captured and manipulated by recombineering paired with primers within the *Zip4* gene itself (Figure 1). Primers flanking the *LoxP* site in intron 5 were used for routine genotyping of the targeted allele in mice (Figure 1). Homologous recombination of the targeting vector into the endogenous locus resulted in the insertion of an *XhoI* site engineered into the *LoxP* sequence in intron 5 which aided in identifying the targeted alleles. Southern blot hybridization was used to screen for the Y-chromosome as described in detail previously [67]. The sequences of oligonucleotides for integration screen and genotyping are shown in Table S1.

### Generation of mice with targeted floxed Zip4 (Slc39a4) alleles

Chimeric mice were generated by microinjection of two independent *Zip4^FX/WT^* ES cell clones into d4 C57BL/6 blastocysts, followed by transfer to pseudopregnant CD-1 foster mothers. Resulting chimeric mice were crossed with C57BL/6 females (Harlan labs). Germline transmission was confirmed by PCR from tail DNA of agouti offspring (Figure 1). *Zip4^FX/WT^* mice were crossed and *Zip4^FX/FX^* offspring were identified by PCR amplification of the *LoxP:XhoI* insertion in intron 5.

### Generation of mice for inducible deletion of Zip4^FX/FX^ (Slc39a4) alleles in the intestinal epithelium

*Zip4^FX/FX^* mice were crossed to create a working colony of mice homozygous for floxed *Zip4* genes. These mice were then crossed with transgenic mice bearing a tamoxifen-dependent Cre recombinase (*vil-Cre-ERT2*: a kind gift from S. Robine, Institut Curie-CNRS, Paris, France) expressed under the control of the *villin* promoter to allow for inducible deletion of the *Zip4* gene specifically in the intestinal epithelium [11] and selected offspring were backcrossed to yield mice heterozygous for *vil-Cre-ERT2* and homozygous *Zip4^FX/FX^*. These mice were then crossed with *Zip4^FX/FX^* mice to yield 50% offspring with *Zip4^FX/FX^*: *vil-Cre-ERT2* alleles and 50% with *Zip4^FX/FX^* alleles. The latter provided age and genetically matched controls for our experiments and were labeled as control (Con) in all the figures.

### Tamoxifen induction of recombination

A tamoxifen stock solution was prepared essentially as described previously [68] by the addition of 100 µl of ethanol to 10 mg of tamoxifen (free base: MP biomedicals, LLC) and heating to 37°C briefly to dissolve the tamoxifen. This solution was then diluted to 1 ml with autoclaved canola oil and heated briefly to 37°C. The stock solution (10 mg tamoxifen/ml) was stored at 4°C for up to two weeks or at −80°C for months. Before injection, the tamoxifen stock solution was heated to 37°C. Adult and recently weaned mice (5 to 8 days post-weaning) were injected I.P. with 100 µl (1 mg tamoxifen) of the tamoxifen stock solution daily for 3 consecutive days unless noted otherwise (e.g. Figure 2F). Neonatal mice (d5 post-partum) were injected I.P. for 5 consecutive days with 10 µl (100 µg) of the tamoxifen stock solution.

### Northern blot hybridization

Total RNA was isolated using Trizol reagent (Invitrogen). Total RNA (3–6 µg) was size-fractionated by agarose-formaldehyde gel electrophoresis, transferred and UV cross-linked to a Zeta Probe GT nylon membrane (BioRad). Northern blot membranes were prehybridized in 0.5 M sodium phosphate, pH 7.0, 7% SDS at 65°C for 30 min and then hybridized in 40% formamide, 0.5 M sodium phosphate, pH 7.0, 7% SDS at 65°C for 24 hr. Membranes were then washed twice in 3X SSC, 0.1% SDS for 30 min each followed by two washes in 1X SSC, 0.1% SDS and two washes in 0.3X SSC, 0.1% SDS for 30 min each at 65°C. Hybrids were detected by autoradiography at −80°C. Duplicate gels were stained with acridine orange to ensure equivalent loading and integrity of total RNA. Riboprobes for mouse *MT-I*, *Zip4*, and *Zip5* [15] and for *IGF-1* mRNAs were as described [69]. Probes were used at 2×10^6^ cpm/ml of hybridization solution.

### Quantitative RT–PCR of *IGF-1* mRNA

Small intestine total RNA was reverse transcribed using Accuscript reverse transcriptase (RT) enzyme (Stratagene) and IGF mRNA and GAPDH mRNA, as an internal control, were amplified using the PerfeCTa Sybr Green FastMix qPCR kit (Quanta Biosciences) and a Miniopticon Real-Time PCR Detection System (BioRad) essentially as described previously [8]. Melting-curve data were collected to confirm PCR specificity. Each cDNA sample was run in triplicate, and the corresponding no-RT sample was included as a negative control. *GAPDH* primers were included in every sample to control for sample variation. *IGF-1* mRNA product in each sample was normalized to that of the *GAPDH* mRNA. The amount of PCR products was measured by threshold cycle (Ct) values and relative mRNA levels were determined as unit values of 2∧[Ct(*GAPDH*) -Ct(*IGF-1*)]. Values presented are fold-change relative to control. The following primers were used: mIGF-1 (s) gctcttcagttcgtgtgtggaccg: mIGF-1 (as) cttctgagtcttgggcatgtcagtgtg. These primers amplify the IGF-1 peptide sequence found in all mIGF-1 variants. GAPDH (s): aggttgtctcctgcgacttca: mGAPDH (as) ccaggaaatgagcttgacaaag.

### Antisera

The following antibodies were used at the indicated dilution for Western blots: Total β-catenin (Cell Signaling: 1∶1000), active β-catenin (Millipore: 1∶1000), p(T172)-AMPK (Cell Signaling: 1∶1000), total AMPK (Cell Signaling: 1∶1000), p(S2448) mTOR1 (Cell Signaling: 1∶1000), total mTOR1 (Cell Signaling: 1∶1000), p(s473)-AKT (Cell Signaling: 1∶1000), total AKT (Cell Signaling: 1∶1000). The following antibodies were used at the indicated dilution for immunohistochemistry (IHC): Sox9 (Millipore: 1∶750), lysozyme (DakoCytomation: 1∶150), phospho-S6 ribosomal protein (Cell Signaling: 1∶400), phosphor (Ser10)-histone H3, (Cell Signaling: 1∶100)

### Histology/cytochemistry/immunohistochemistry (IHC)

The first ∼5 cm of small intestine (3 to 5 mice per group) was collected, flushed with cold PBS, cut into small pieces and fixed in Bouin's fixative or 4% paraformaldehyde in PBS overnight at 4°C. Fixed tissues were embedded in paraffin and sections (1 µm) were prepared by Histo-Scientific Research Laboratories (HSRL). Bouin's fixed sections were deparaffinized, rehydrated and stained with hematoxylin-eosin for examination of gross morphology. Paraformaldehyde fixed tissues were deparaffinized, rehydrated and stained using the Periodic Acid-Schiff (PAS) staining system (Sigma-Aldrich) to detect mucin (mainly Muc2 and Muc3) and identify goblet cells. Serial sections were pretreated with α-amylase (239 µg/ml for 1 hr at 37°C: Worthington Biochemical Corporation) to test for glycogen contribution to the PAS staining. For IHC, paraformaldehyde fixed serial sections were deparaffinized, rehydrated and antigens were retrieved in 10 mM citrate, pH 6.0, using a 2100 Retriever pressure cooker (PickCell Laboratories). Proteinase K digestion (20 µg/ml in 50 mM Tris base, pH 8.0, 0.1 mM EDTA, 0.5% Triton X- 100 at 37°C for 10 min) was used for antigen retrieval for the lysozyme antibody. Samples were processed using the Histostain Plus LAB SA Detection System (Invitrogen) according to the manufacturer's instructions using the antibodies listed above. Stained slides were counterstained briefly in Mayer's hematoxylin (Sigma) and photographed using a Leica DM 4000B microscope (Leica-microsystems) with Adobe Photoshop image capture software (Adobe).

### Detection of Paneth cell zinc

Paneth cell labile zinc was detected using the zinc-specific-responsive fluorescent dye Zinpyr-1(ZP1) (Millitech) essentially as described with modifications [26]. The small intestine from 3 to 5 mice per group was isolated, flushed with cold PBS and cut into small pieces that were dropped into cold 0.6 M sucrose in PBS. Once the small intestine pieces sank (1 to 2 min) they were transferred to a solution of 2 parts 0.6 M sucrose plus 1 part TFM embedding compound (Triangle Biomedical Sciences, Inc.) until they sank (2 to 3 min). The intestine pieces were then immersed in TFM, frozen in dry ice-propanol and 6 µm frozen sections were cut. Frozen sections were thawed for ∼30 seconds at room temperature in a solution containing 20 µM ZP1 and 0.5 µM DAPI in 147 mM NaCl, 4 mM KCl, 3 mM CaCl_2_, 0.9 mM MgCl_2_, 11 mM HEPES, pH 7.4, and 10 mM glucose, washed briefly in PBS and then photographed using a Leica DM 4000B microscope (Leica-microsystems) with Adobe Photoshop image capture software (Adobe). ZP1 was visualized using the +L5 (Leica-microsystems) GFP filter cube and DAPI stained nuclei were visualized using the +A4 filter cube (Leica-microsystems). Brightness of the photographs was reduced in Photoshop.

### Western blot analysis

Liver proteins were isolated by homogenizing ∼100 mg of tissue in 400 µl of RIPA buffer (150 mM NaCl, 50 mM Tris base, pH 8.0, 1% NP40, 0.5% deoxycholate, 0.1% SDS, plus Complete Protease Inhibitor Cocktail and PhosStop phosphatase inhibitor cocktail (Roche Applied Science). Tissues were homogenized at 4°C for 20 strokes using a teflon-glass homogenizer. Samples were then centrifuged at 10,000× g for 10 min at 4°C, the supernatant was collected and protein concentration was determined using BCA reagent (Pierce Biotech). Proteins (30 µg) were resolved on 7.5% or 10% SDS-polyacrylamide gels and transferred to polyvinylidene difluoride membranes (Life Science Products, Inc.) using a Transblot semi-dry transfer apparatus (BioRad) according to the manufacturer's instructions. Membranes were blocked overnight in 5% milk in PBS-T or 5% BSA in PBS-T as suggested by the antibody source and then incubated with primary antibody in blocking solution at the appropriate dilution for 2 h at room temperature. After extensive washing, membranes were incubated with goat anti-rabbit horseradish peroxidase-conjugated secondary antibody (1∶2000) and the blot was developed using ECL Plus reagent (Amersham Biosciences) according to manufacturer's instructions and detected using Kodak BioMax MS film (Kodak).

### Elemental and essential metal determination

Elemental profiling via inductively coupled plasma mass spectrometry (ICP-MS) was performed at the Purdue University Ionomics Facility, Purdue University, West Lafayette, Indiana as described previously [21]. The following elements were measured: Na, Mg, P, K, Ca, Fe, Co, Cu, Zn, Mn, As, Se, and Mo. Mouse tissues (n = 5 to 10: <100 mg wet weight each sample) were dried at 95°C in a vacuum oven. Dried samples of about 5 to 10 mg were weighed into Pyrex tubes and digested in 0.7 to 1.5 ml concentrated HNO_3_ at 110°C for 4 h. Each sample was diluted as optimal with 18 MOhm water and analyzed on an Elan DRCe ICP-MS (PerkinElmer). Methane was used as a collision cell gas to measure iron. Gallium and indium were used as internal standards, added to the digestion acid bottle to a concentration of 20 µg l^−1^. National Institute of Standards and Technology traceable single element ICP standards (Ultrasci) were used to make up the calibration standards. Tissue concentrations were determined as µg g^−1^ dry weight (ppm) of each element.

### Statistical analyses

Graphs were generated and statistical analyses performed using GraphPad Prism5 software (GraphPad Software). Statistical significance was determined using the Unpaired T-test (two-tailed) and values were considered different if P<0.05. Data are expressed as the mean ± S.E.M. *indicates P<0.05; *** indicates P<0.001; **** indicates P<0.0001

## Supporting Information

Figure S1 The intestine *Zip4* gene controls growth and viability. (A) Neonatal mice homozygous for the floxed *Zip4* gene and positive for the *vil-CreERT2* gene (*Zip4* intest KO) and littermates homozygous for the floxed *Zip4* gene but negative for the *vil-CreERT2* gene (Con) were injected for 5 consecutive days with tamoxifen beginning 5 days post-partum. After weaning on day 21 their body weights were measured daily for three days. These mice were fed normal chow after weaning. (B) Recently weaned mice (*Zip4* intest KO and Con) were injected for 3 consecutive days with tamoxifen and their body weight was measured daily. Every *Zip4*-intestine KO mouse examined died within 16 days of initiation of the knockout. (C) Recently weaned mice (Zip4 intest KO and Con) were given a single injection of tamoxifen and their body weights were measured daily for 26 days.
(TIF)

Figure S2 Intestine-specific deletion of *Zip4* does not apparently cause a rapid change the distribution or abundance of goblet and enteroendocrine cells in the villi of the small intestine. Recently weaned mice were injected 3 consecutive days with tamoxifen and the small intestines from *Zip4*-intestine knockout (*Zip4* intest KO) mice (5 mice per group) were harvested on the indicated days after initiation of the knockout. Paraffin sections of small intestine were processed for IHC using an antibody against mouse Sox9 (Sox9). Brown color indicates positive immunostaining. An arrow points to Sox9 positive enteroendocrine cells interspersed among the villus enterocytes. Serial sections were also stained with PAS to detected mucin positive goblet cells. Dark pink to red color indicates positive PAS staining. An arrow points to mucin positive goblet cells interspersed among the villus enterocytes.
(TIF)

Table S1 List of oligonucleotides used for integration screening in embryonic stem cells and genotyping of *Zip4* alleles in mice.
(DOCX)
