# Supplementary figures and images for: A Mouse Model of Acrodermatitis Enteropathica: Loss of Intestine Zinc Transporter ZIP4 (Slc39a4) Disrupts the Stem Cell Niche and Intestine Integrity
Source: PLoS Genet. 2012 Jun 21;8(6):e1002766. doi: 10.1371/journal.pgen.1002766 (PMC3380849; doi:10.1371/journal.pgen.1002766)

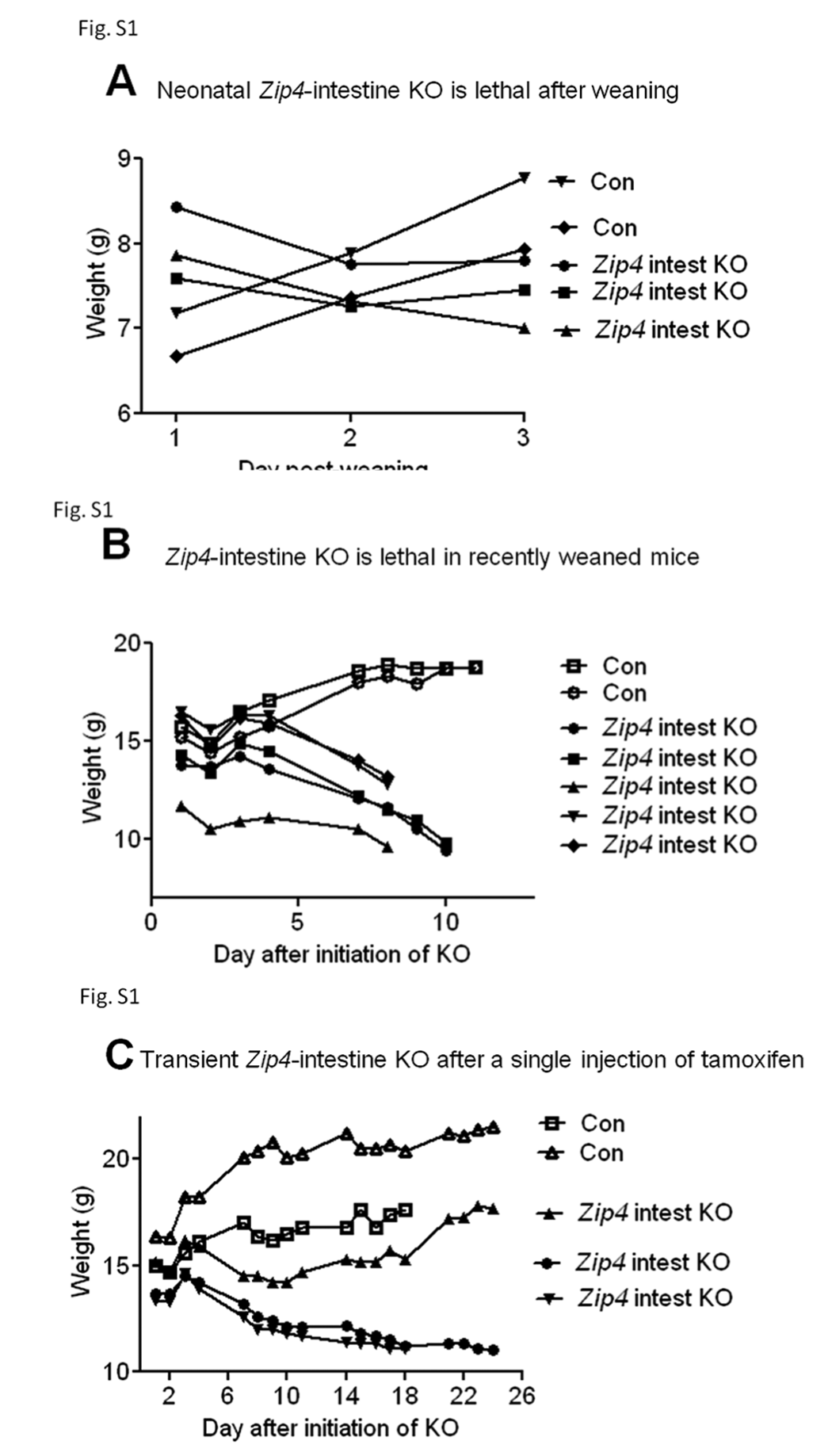

Supplement: Figure S1 — The intestine Zip4 gene controls growth and viability. (A) Neonatal mice homozygous for the floxed Zip4 gene and positive for the vil-CreERT2 gene (Zip4 intest KO) and littermates homozygous for the floxed Zip4 gene but negative for the vil-CreERT2 gene (Con) were injected for 5 consecutive days with tamoxifen beginning 5 days post-partum. After weaning on day 21 their body weights were measured daily for three days. These mice were fed normal chow after weaning. (B) Recently weaned mice (Zip4 intest KO and Con) were injected for 3 consecutive days with tamoxifen and their body weight was measured daily. Every Zip4-intestine KO mouse examined died within 16 days of initiation of the knockout. (C) Recently weaned mice (Zip4 intest KO and Con) were given a single injection of tamoxifen and their body weights were measured daily for 26 days. (TIF) [file pgen.1002766.s001.tif]
