# Supplementary material for: A Mouse Model of Acrodermatitis Enteropathica: Loss of Intestine Zinc Transporter ZIP4 (Slc39a4) Disrupts the Stem Cell Niche and Intestine Integrity
Source: PLoS Genet. 2012 Jun 21;8(6):e1002766. doi: 10.1371/journal.pgen.1002766 (PMC3380849; doi:10.1371/journal.pgen.1002766)
Supplement: Table S1 — List of oligonucleotides used for integration screening in embryonic stem cells and genotyping of Zip4 alleles in mice. (DOCX) [file pgen.1002766.s003.docx]

**Table S1**

| **Primer application** | **Sense** | **Antisense** |
| --- | --- | --- |
| 5’ integration screen | caggaagccaaggctatacagagaaaccctgtctcc | cgttggtaatgttttgactcgagacaaggacccc |
| 3’ integration screen | ggggtccttgtctcgagtcaaaacattaccaacg | cctggaactcagagatctgcctgcctgaagtc |
| Genotyping screen preCRE | aggaggaagagtagtggatttcaagg | cgagccatagagataccctgtgg |
| Genotyping screen postCRE* | aggaggaagagtagtggatttcaagg | cttttctggattcatcgactgtgg |

*PCR reaction contained three primers: PreCRE antisense, PostCRE sense and antisense
